# Integrated assessment of the clinical and biological value of ferroptosis-related genes in multiple myeloma

**DOI:** 10.1186/s12935-022-02742-4

**Published:** 2022-10-23

**Authors:** Bibo Fu, Ruonan Shao, Huizhong Wang, Guanjun Chen, Shenrui Bai, Hua Wang

**Affiliations:** Department of Hematologic Oncology, State Key Laboratory of Oncology in South China, Collaborative Innovation Center for Cancer Medicine, Sun Yat-sen University Cancer Center, 651 Dongfeng Road East, Guangzhou, 510060 Guangdong People’s Republic of China

**Keywords:** Ferroptosis, Prognostic model, Immune infiltration, Therapy, Multiple myeloma

## Abstract

**Background:**

Ferroptosis is an iron-dependent mode of cell death that could be induced by erastin and exert antitumor effects. However, the clinical and biological roles of ferroptosis-related gene (FRG) signature and the therapeutic value of erastin in multiple myeloma (MM) remained unknown.

**Methods:**

Clinical and gene expression data of MM subjects were extracted from the Gene Expression Omnibus (GEO) public database. Univariable cox analysis was applied to determine FRGs related to survival and the least absolute shrinkage and selection operator (LASSO) regression analysis was used to develop a prognostic model. Prediction accuracy of the model was estimated by receiver operating characteristic (ROC) curves. Functional pathway enrichments and infiltrating immune status were also analyzed. We conducted in vitro experiments to investigate the combination therapy of erastin and doxorubicin.

**Results:**

17 FRGs were strongly associated with patient survival and 11 genes were identified to construct the prognostic model. ROC curves indicated great predictive sensitivity and specificity of the model in all cohorts. Patients were divided into low- and high-risk groups by median risk score in each cohort and the survival of the low-risk group was significantly superior than that of the high-risk group. We also observed a close relevance between functional pathways and immune infiltration with risk scores. Moreover, we combined erastin and doxorubicin in our in vitro experiments and found synergetic antitumor effects of the two agents, and the underlying mechanism is the overgeneration of intracellular Reactive Oxygen Species (ROS).

**Conclusions:**

We demonstrated the important value of ferroptosis in patient prognosis and as a potential antitumor target for MM.

**Supplementary Information:**

The online version contains supplementary material available at 10.1186/s12935-022-02742-4.

## Introduction

Multiple myeloma (MM) is a malignancy derived from plasma cells, accounting for roughly 1% of neoplastic diseases [1]. The 5-year overall survival (OS) of MM is about 50%. However, with the application of novel therapies including proteasome inhibitors and immunotherapy, the prognosis of MM has greatly improved [2]. Several prognostic models incorporated with clinical parameters or cytogenetic aberrations, such as the International Staging System (ISS) and the Revised International Staging System (R-ISS), were traditionally applied in risk stratification of MM [3, 4]. However, there is complex mechanism under the pathogenesis of MM, and some molecular changes are closely associated with the occurrence and clinical outcomes of the disease [5, 6]. Identifying novel biomarkers for new prognostic models is conductive to better risk stratification and targeted treatment. Recently, more and more studies have shown that prognostic models based on gene signature exhibited superior survival prediction in various malignancies, including hematological neoplasms [7, 8].

Ferroptosis, first proposed in 2012, is an iron-dependent mode of cell death characterized by the disorder of lipid and iron metabolism [9]. The occurrence of lipid peroxidation and the accumulation of intracellular iron leads to the excessive production of reactive oxygen species (ROS), which induces cell damage and inhibits tumor growth [9, 10]. Inducing ferroptosis has emerged as a promising therapy for neoplastic diseases, especially those resistant to conventional chemotherapy regimens [11–13]. In addition, numerous genes were reported as important regulator of ferroptosis, and a large number of data have shown that ferroptosis-related gene (FRG) signature could accurately predict survival outcomes of various malignancies, such as hepatocellular carcinoma, breast cancer, bladder cancer and glioma [7, 14–16]. However, there were few data about the role of ferroptosis in myeloma. To our knowledge, no FRG prognostic model has been developed for MM. In this study, we identified FRGs that closely associate with prognosis of MM patients and constructed a prognostic model with remarkable prediction accuracy. We also explored the gene enriched pathways and the relevance between the model and tumor immunity. The prognostic model provides a promising prospect for the diagnosis, survival prediction and novel therapeutic strategies of MM.

## Methods

### Data acquisition

The mRNA expression levels and clinical data of MM subjects were retrieved from 3 datasets in the GEO database (http://www.ncbi.nlm.nih.gov/geo/). GSE136337 was retrieved as the training cohort, while GSE24080 and GSE57317 the external validation cohorts. In addition, differential gene expression information between normal and tumor tissues was obtained from GSE6477 and GSE118985. Totally, 213 known FRGs through the FerrDb database (http://www.zhounan.org/ferrdb/) [17] or reported by relevant literatures [18–20] were acquired for subsequent analyses. All data were available in public database and ethical approval was not required.

### Prognostic model construction and validation

Univariable cox regression analysis was performed to determine the FRGs significantly related to MM survival with *P* < 0.05. Then, we apply the least absolute shrinkage and selection operator (LASSO) analysis with “glmnet” R package to construct a prognostic gene model. The quintessential penalty parameter λ of the model were determined according to the minimum criterion through tenfold cross verification. The risk score of a subject was equal to the sum of the expression level of selected genes multiplied by the corresponding weighting coefficients. Survival analyses between different risk groups were accessed by Kaplan–Meier curves. Receiver operating characteristic (ROC) curves and areas under ROC curves (AUROC) were employed to estimate prediction accuracy.

### Functional analysis

Functional pathways were explored by the GSEAv4.0.2 software (http://software.broadinstitute.org/gsea/login.jsp) using the c2.cp.kegg.v7.0.symbols gene sets. Statistical significance was defined as NOM *P* < 0.05. We accessed the protein interaction network by Gene cloud biotechnology information (GCBI) and used the cBioPortal for Cancer Genomics (http://www.cbioportal.org/) to study FRGs mutant profile in the Cancer Cell Line Encyclopedia database (CCLE, https://portals.broadinstitute.org/ccle). Infiltration scores of immune cells and molecular pathways were computed by single-sample gene set enrichment analysis (ssGSEA) [21].

### Cell lines and agents

The well-established MM cell lines H929 and RPMI-8226 were obtained from the American Type Culture Collection (ATCC, Manassas, VA, USA) and cultured at 37 ℃, 5% CO_2_ humid incubator with RPMI1640 medium (ESscience, Shanghai, China) supplemented by 10% FBS, penicillin and streptomycin each 100 IU/mL. Erastin and doxorubicin were purchased from Selleck. Erastin was dissolved in dimethyl sulfoxide (DMSO) to 10 mM and doxorubicin was dissolved in culture medium to 1 mM, and then both were stored at −80 ℃.

### Cell viability and cell death assays

Cells were planted in 96-well plates with 8000 cells per well and treated with specified concentration of each group of agents for 48 h. Then, the cell counting kit-8 (CCK8; APExBIO, America) was used to measure cell viability. According to the manufacture’s instruction, cells were added with 10 μL/well CCK8 reagent. After incubated at 37 ℃ for 2–3 h, values at 450 nm was recorded with a microplate reader. Besides, we treated cells with single or combined agents for 48 h and performed 5‐ethynyl‐2′‐deoxyuridine (EdU) proliferation assay (Beyotime, Nanjing, China) following manufacture’s protocol. The cell proliferation was visually observed under fluorescence microscopy.

Cell death was detected with Annexin V-FITC/PI assay kit (ESscience, Shanghai, China). After 48 h pre-treatment of agents, cells were collected and washed twice in pre-chilled PBS. With cells resuspended in 500 μL binding buffer and stained by Annexin V-FITC/PI in the dark for 10–15 min according to directions, detections were progressed by flow cytometry.

### ROS assay

The intracellular Reactive Oxygen Species (ROS) level was tested by DCFH-DA fluorescent probe of ROS assay kit (Solarbio, Guangzhou, China). Cells pre-treated with specified agents for 48 h were harvested at 1000 rpm, 5 min and loaded with fluorescent probes. Results were measured by fluorescence microscopy and fluorescence microplate reader.

### Western blot

After incubating with different groups of drugs for 48 h, the cells were collected and protein was extracted with RIPA lysis buffer supplemented with protease and phosphatase inhibitors. Then the protein was separated by SDS-PAGE electrophoresis, transferred to polyvinylidene fluoride (PVDF) membranes and incubated with the corresponding primary antibody (β-actin, GPX4, SLC7A11, Keap1, KRAS, ERK, Raf) and peroxidase-conjugated secondary antibody.

### ATP assay

Cells were collected after pre-treatment and ATP were extracted and measured by a firefly luciferase-based ATP assay kit (Beyotime, Nanjing, China) according to manufacturer’s protocol.

### Statistical analysis

The differences of OS between low- and high-risk groups were compared by Kaplan–Meier curves and log-rank tests with “survival” R package. Univariable and multivariable cox regression was applied to determine independent prognostic factors with “survminer” R package. All statistical analysis was performed using R software (version 3.6.1). The combination indexes (CI) were calculated by CompuSyn software [22]. CI values < , = , or > 1 represent synergistic, mean additive or antagonistic effects of agents, respectively. Statistical significance was defined with a two-sided *P* < 0.05.

## Results

### Selection of cohorts and baseline characteristics

1040 patients in 3 cohorts with available gene expression and survival information were utilized in the study. The training cohort from GSE136337 dataset for developing the prognostic gene model and one external validation cohort from GSE24080 dataset had adequate data of baseline clinical characteristics, while another validation cohort from GSE57317 dataset did not. The baseline characteristics were shown in Table 1.

**Table 1:** Baseline characteristics of the training and validation cohorts

Characteristics	Training cohort GSE136337 (n = 426)	Validation cohort GSE24080 (n = 559)	Validation cohort GSE57317 (n = 55)
Gender			
Male	261 (61%)	337 (60%)	–
Female	165 (39%)	222 (40%)	–
Age (years)			
≤ 65	307 (72%)	432 (77%)	–
> 65	119 (28%)	127 (23%)	–
Albumin (g/dL)			
< 3.5	88 (21%)	77 (14%)	–
≥ 3.5	337 (79%)	482 (86%)	–
NA	2	–	–
β2M (mg/L)			
< 3.5	187 (45%)	319 (57%)	–
3.5–5.4	111 (26%)	120 (22%)	–
≥ 5.5	121 (29%)	119 (21%)	–
NA	7	1	–
LDH (U/L)			
≤ 250	398 (94%)	509 (91%)	–
> 250	24 (6%)	50 (9%)	–
NA	4	–	–
ISS			
I	168 (40%)	294 (53%)	–
II	135 (32%)	144 (26%)	–
III	121 (28%)	120 (21%)	–
NA	2	1	–
RISS			
I	83 (20%)	–	–
II	270 (64%)	–	–
III	66 (16%)	–	–
NA	7	–	–
Risk			
Low	213 (50%)	280 (50%)	28 (51%)
High	213 (50%)	279 (50%)	27 (49%)
Transplant(s)			
< 3	331 (78%)	–	–
≥ 3	95 (22%)	–	–
Del(13q)			
TRUE	77 (18%)	–	–
FALSE	349 (82%)	–	–
Del(11q)			
TRUE	8 (2%)	–	–
FALSE	418 (98%)	–	–
Del(17p)			
TRUE	15 (4%)	–	–
FALSE	411 (96%)	–	–
Del(16q)			
TRUE	14 (3%)	–	–
FALSE	412 (97%)	–	–
Del(1p32)			
TRUE	85 (20%)	–	–
FALSE	341 (80%)	–	–
Myc(8q24)			
TRUE	20 (5%)	–	–
FALSE	406 (95%)	–	–
*t* (11,14)			
TRUE	22 (5%)	–	–
FALSE	404 (95%)	–	–
*t* (12,14)			
TRUE	1 (1%)	–	–
FALSE	425 (99%)	–	–
*t* (14,16)			
TRUE	1 (1%)	–	–
FALSE	425 (99%)	–	–
*t* (14,20)			
TRUE	1 (1%)	–	–
FALSE	425 (99%)	–	–
Hyperdiploid			
TRUE	85 (20%)	–	–
FALSE	341 (80%)	–	–
Survival status			
Alive	243 (57%)	387 (69%)	43 (78%)
Dead	183 (43%)	172 (31%)	12 (22%)

### Development and validation of the prognostic gene model

**Fig. 1:**
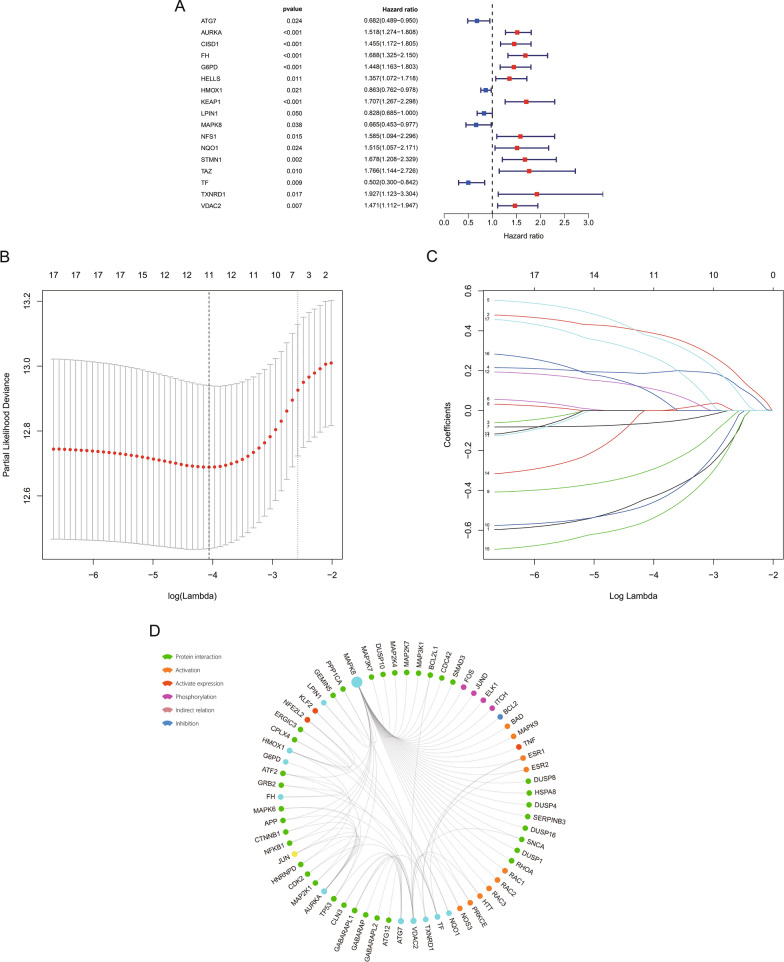
Development of the prognostic model based on FRGs in the training cohort. **A** A forest plot showing the FRGs associated with OS according to univariable cox analysis. **B** Variable selection by lasso regression analysis with 1000 bootstrap replicates. **C** LASSO coefficients of FRGs. **D** The protein–protein interactions among the model related proteins and the other proteins. Green, protein interaction; orange, activation; red, activate expression; purple, phosphorylation; grey pink, indirect relation; blue, inhibition. The circle size represents the number of interacting proteins that are linked to a specific protein (the bigger the circle size, the more interacted proteins)

**Fig. 2:**
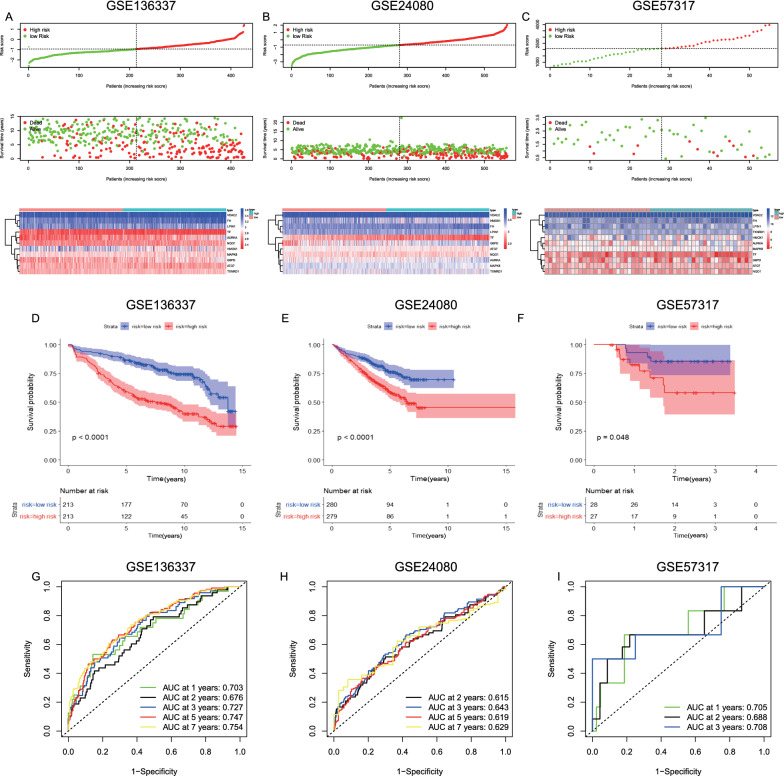
Validation of the prognostic model based on FRGs. **A**–**C** Risk score distribution and survival status; heat maps showing the distribution of risk scores under different gene expression in MM. **D**–**F** K-M curves indicated a superior survival of the low-risk group than the high-risk group. **G**–**I** Time-dependent ROC curves used to measure OS prediction accuracy of the prognostic model

17 FRGs closely associated with OS of MM patients in the GSE136337 dataset were identified by univariable cox analysis (Fig. 1A). Next, the lasso regression analysis was applied to choose the 11 optimal genes related to prognosis and develop the prognostic model (Additional file 1: Table S1, Fig. 1B and C). Comparisons of the expression level of these genes in normal and tumor tissues were displayed in Additional file 2: Figure S1. ATG7, AURKA, HMOX1 and TF showed a lower expression while VDAC2 showed a higher expression in MM than normal tissue in both GSE6477 and GSE118985 datasets. The proteins potentially related with the model and protein interactions were studied by GCBI analysis (Fig. 1D). The expression and mutation profile of these genes by cBioPortal analysis was displayed in Additional file 3: Figure S2. The following formula is for calculating the risk scores: risk score = (−0.4358 × expression level of ATG7) + (0.3907 × expression level of AURKA) + (0.1889 × expression level of FH) + (0.3740 × expression level of G6PD) + (−0.0624 × expression level of HMOX1) + (−0.2988 × expression level of LPIN1) + (−0.4663 × expression level of MAPK8) + (0.1110 × expression level of NQO1) + (−0.5460 × expression level of TF) + (0.0926 × expression level of TXNRD1) + (0.2743 × expression level of VDAC2). Higher risk scores were related to worse clinical presentations in the training cohort (Additional file 4: Figure S3). We applied the median risk scores in the training (Fig. 2A) and two validation (Fig. 2B and C) cohorts to separate patients into low- and high-risk groups. The 5-year OS of the low-risk group (86.8%, 95% confidence interval CI 82.3–91.3%) was significantly superior than that of the high-risk group (58.9%, 95% CI 52.2–65.6%, *P* < 0.0001) in the training cohort (Fig. 2D). Significant differences in survival between low- and high-risk groups were also observed in two validation cohorts (*P* < 0.05, Fig. 2E and F). Time-dependent ROC analysis showed that AUROCs in the training cohort (Fig. 2G) at 1-, 2-, 3-, 5- and 7-year were 0.703, 0.676, 0.727, 0.747 and 0.754, respectively, which indicated great sensitivity and specificity in survival prediction of the gene model. Comparing AUROC results in the validation cohorts were shown in Fig. 2H and I.

### Establishment and validation of the predictive nomogram

**Fig. 3:**
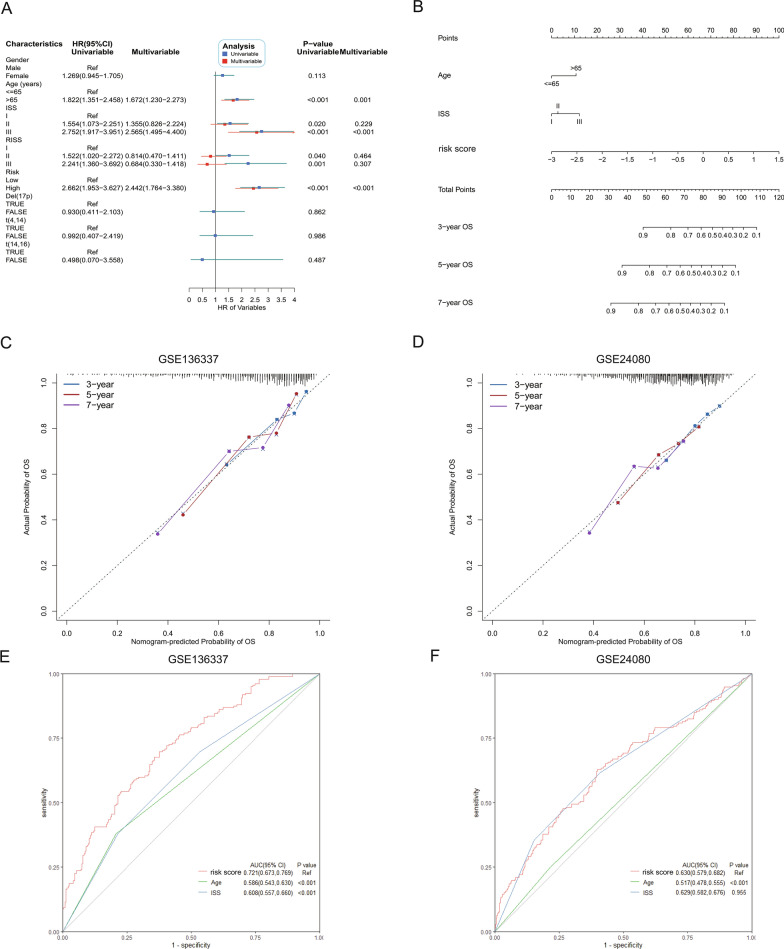
Establishment and validation of a predictive nomogram. **A** Univariable and multivariable cox analyses to determine the independent predictors for OS. **B** The nomogram for 3-, 5-, 7-year OS prediction. **C**–**D** Calibration plots for 3-, 5-, 7-year OS prediction of the nomogram. **E**–**F** ROC curves for comparing prediction accuracy among independent predictors of the nomogram

We included several baseline clinical characteristics and risk score into univariable and multivariable cox analyses to identify the independent prognostic factors in the training cohort. In the univariable analysis, higher age, ISS, RISS and risk score were associated with worse OS. Age, ISS and risk score were still independent predictors of OS in the multivariable analysis (Fig. 3A) and were used to establish the nomogram shown in Fig. 3B. Calibration plots showed excellent consistency of the nomogram-predicted and actual probability of 3-, 5-, 7-year OS in both training and the GSE24080 validation cohorts (Fig. 3C and D). The AUROC of the risk score was 0.721 (95% CI 0.673–0.769; Fig. 3E) in the training cohort, which was significantly higher than that of age (0.586, 95% CI 0.543–0.630; *P* < 0.001) and ISS (0.608, 95% CI 0.557–0.660; *P* < 0.001). In the GSE24080 cohort, AUROC of the risk score (0.630, 95% CI 0.579–0.682; Fig. 3F) was significantly higher than that of age (0.517, 95% CI 0.478–0.555; *P* < 0.001).

### Functional analysis in 3 cohorts

**Fig. 4:**
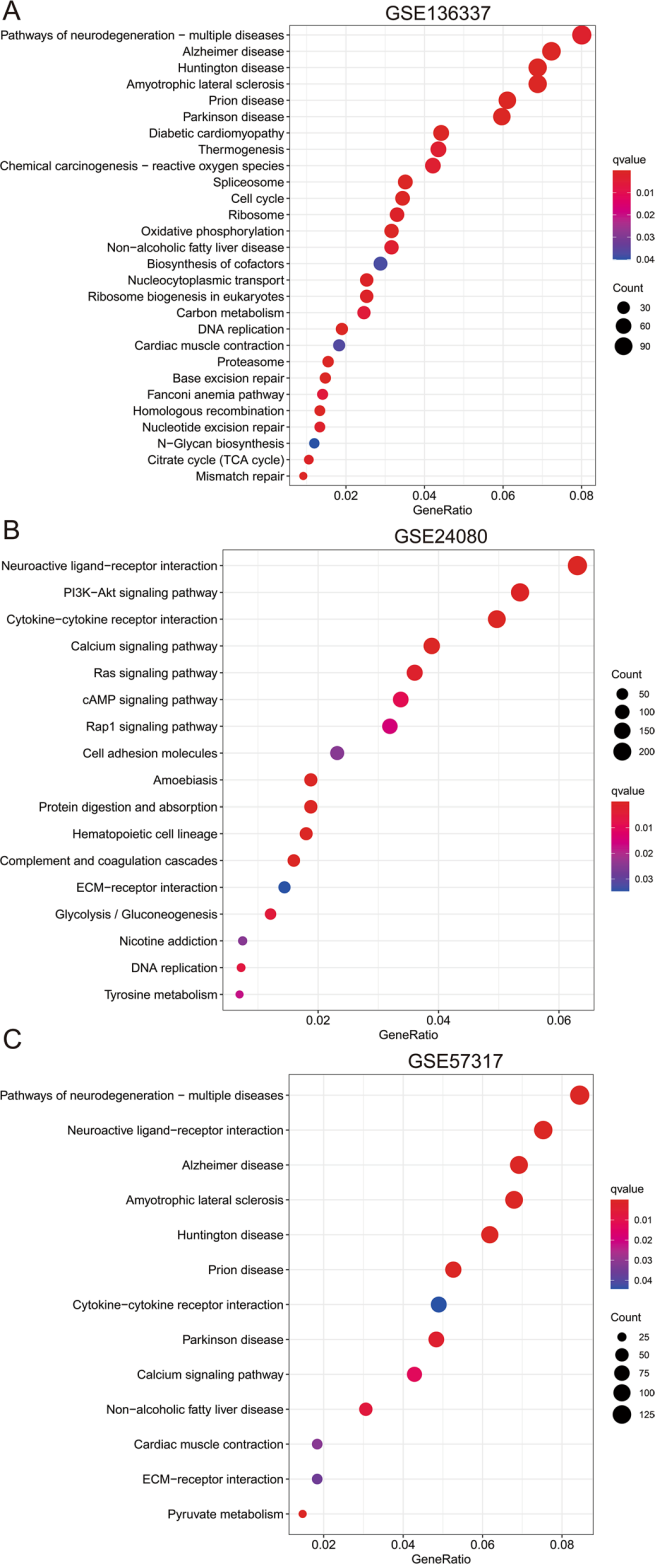
**A**–**C** The most significant enrichment of KEGG pathways in three cohorts

**Fig. 5:**
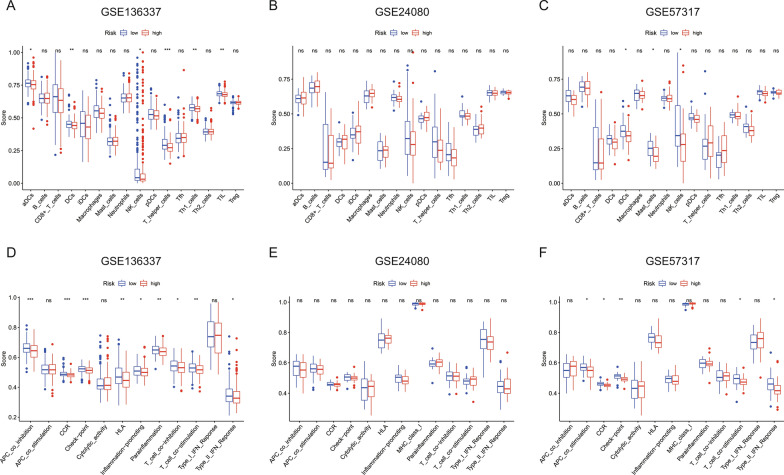
Comparison of ssGSEA scores between different risk groups in three cohorts. Boxplots exhibiting the scores of 16 immune cells **A**–**C** and 13 immune-related functions **D**–**F**

We performed GSEA analysis to identify the biological functions and signaling pathways related to risk score. Several biological processes and pathways that associated with ferroptosis such as oxidative phosphorylation and RAS signaling pathway were enriched in 3 cohorts (Fig. 4A, B and C). In addition, some immune-related cellular functions and molecular signaling were identified in the GSE24080 and GSE57317 cohorts, which include complement and coagulation cascades, cell adhesion molecules, hematopoietic cell lineage, cytokine-cytokine receptor interaction and rap1 signaling pathway (Fig. 4B and C). Therefore, ssGSEA was conducted to investigate the association of immune cell (Fig. 5A, B and C) and immune function (Fig. 5D, E and F) enrichment with risk score. In the training cohort, the enrichment scores of aDCs, DCs, NK_cells, T_helper_cells, Th1_cells and TIL in low-risk group were significantly different from that in high-risk group. In the GSE57317 validation cohort, the low-risk group has higher enrichment scores of DCs, Mast_cells and NK_cells than the high-risk group. The scores of CCR, Check-point, T_cell_co-stimulation and Type_II_IFN_Reponse were significantly different between the low- and high-risk group from both training and GSE57317 validation cohort. However, no difference of immune status was observed between the two groups in GSE24080 cohort.

### Combination of erastin and doxorubicin synergistically inhibited cell proliferation, induced cell death and stimulated ROS accumulation

**Fig. 6:**
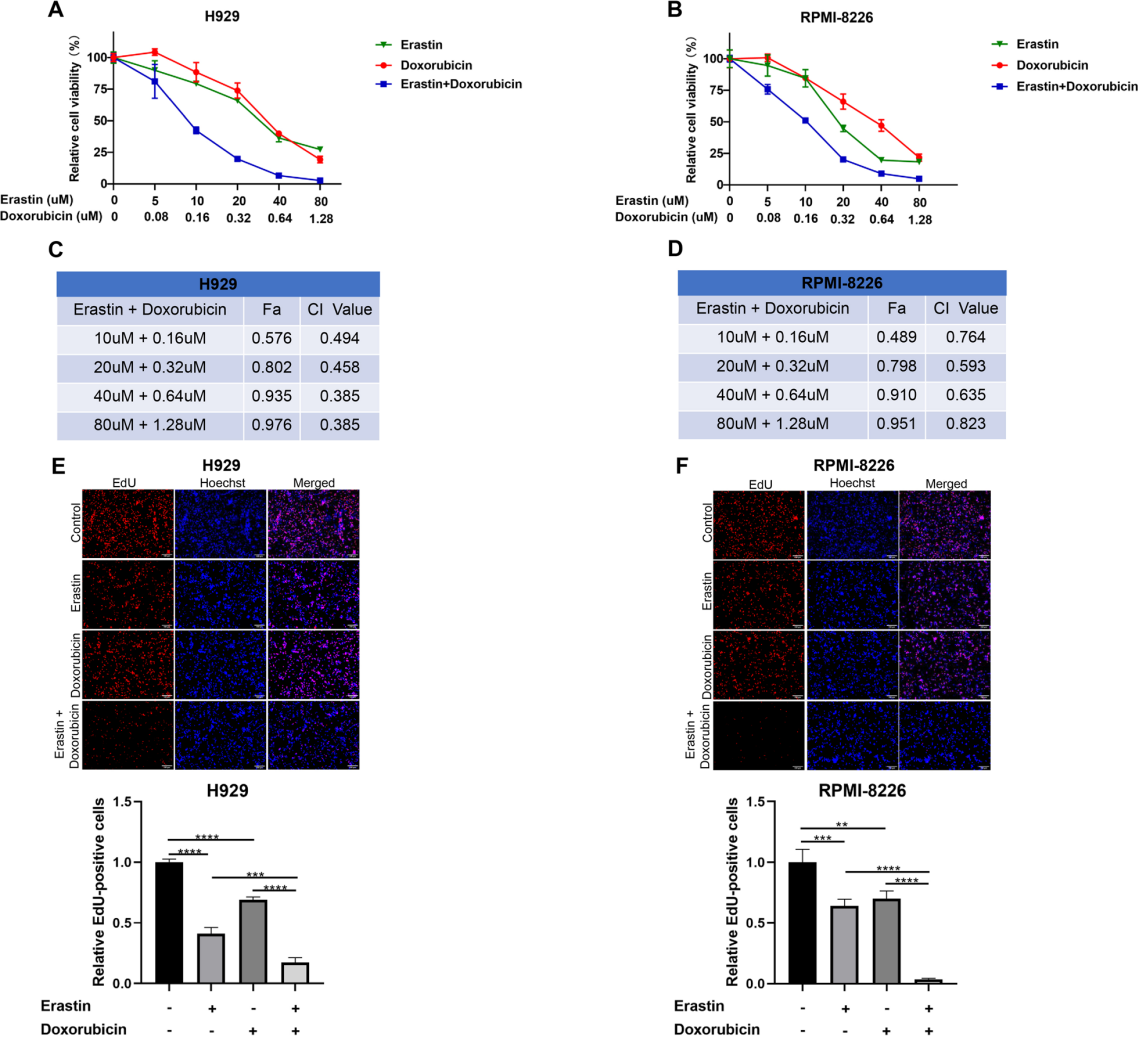
Synergistic effects in inhibiting cell proliferation of the agents. Constant ratio analysis indicated that combination treatment synergistically inhibited cell viability as measured by CCK8 assay after pre-treating H929 **A** and RPMI-8226 **B** cell lines with specified concentration of erastin and/or doxorubicin for 48 h. **C**–**D** Fraction-affected (Fa) and CI are explored after 48 h pre-treatment with erastin and doxorubicin combination, CI < 1 represents synergy. EdU results observed with fluorescence microscopy after **E** H929 cells were treated with DMSO, erastin (15 μM) and/or doxorubicin (0.2 μM) and **F** RPMI-8226 cells were treated with DMSO, erastin (5 μM) and/or doxorubicin (0.3 μM) for 48 h. scale bar = 100 μm

**Fig. 7:**
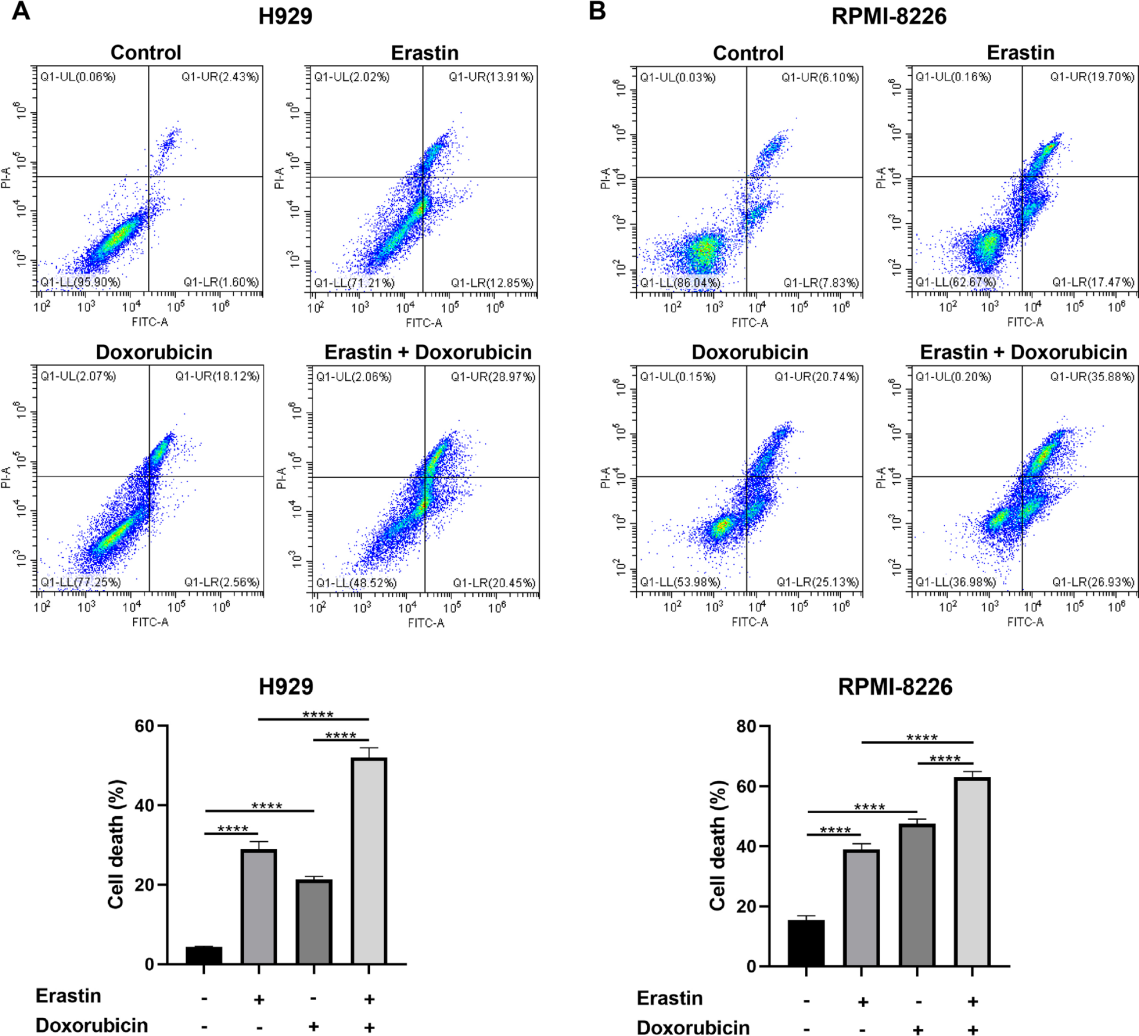
Cell death measured by flow cytometry after **A** H929 cells were treated with DMSO, erastin (15 μM) and/or doxorubicin (0.2 μM) and **B** RPMI-8226 cells were treated with DMSO, erastin (5 μM) and/or doxorubicin (0.3 μM) for 48 h

**Fig. 8:**
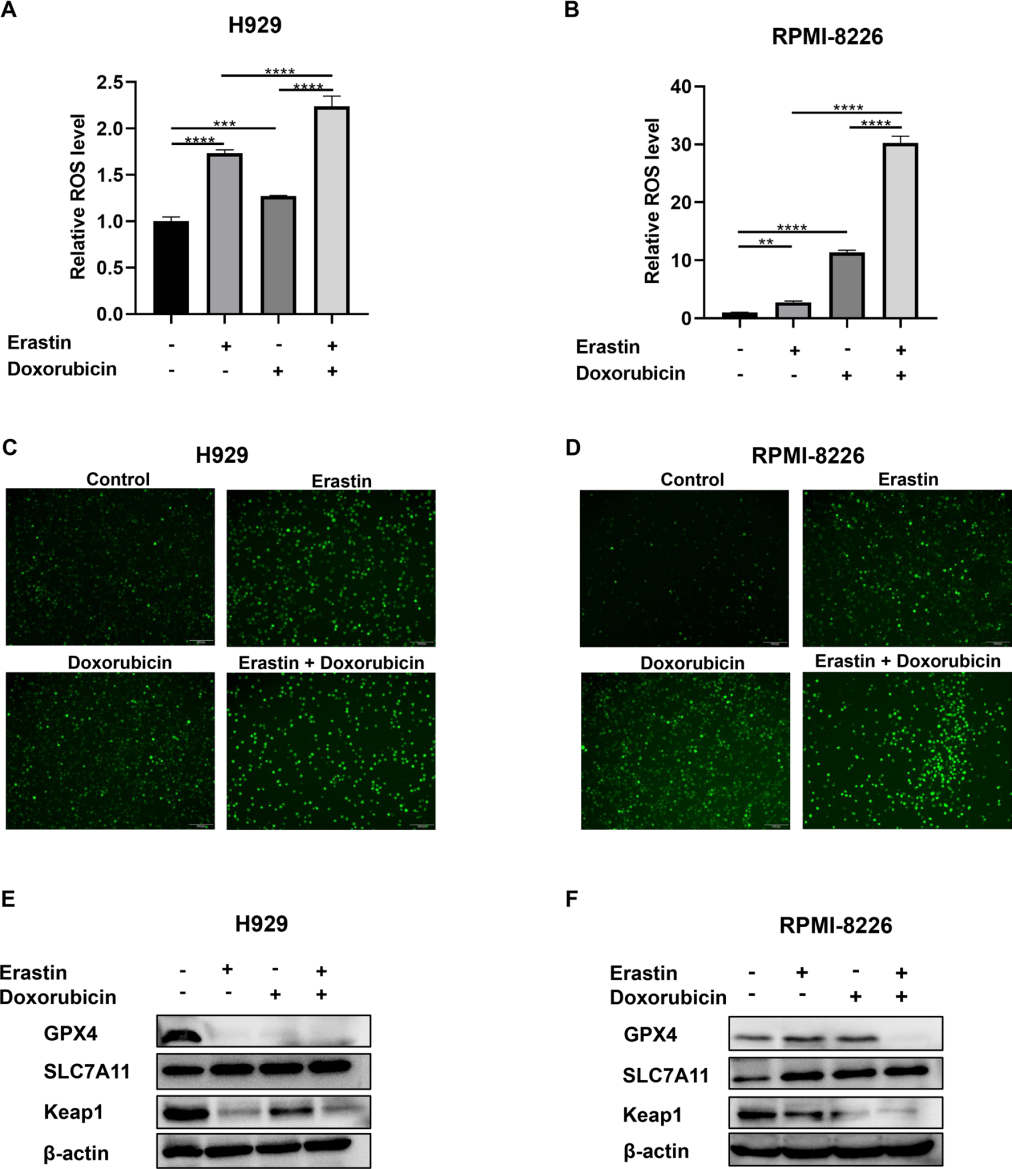
Intracellular ROS production assessed by fluorescence microplate reader **A**–**B** or fluorescence microscopy **C**–**D** after H929 cells were treated with DMSO, erastin (15 μM) and/or doxorubicin (0.2 μM) and RPMI-8226 cells were treated with DMSO, erastin (5 μM) and/or doxorubicin (0.3 μM) for 48 h. The protein level of GPX4, SLC7A11 and Keap1 **E**–**F** analyzed by western blot after H929 or RPMI-8226 cells were treated with DMSO, erastin and/or doxorubicin

Erastin is a well-studied ferroptosis inducer that works by binding to VDAC2 to induce excessive ROS production. Co-treatment of erastin and doxorubicin synergized in inhibition of cell proliferation and induction in cell death. The IC_50_ values of erastin in H929 and RPMI-8226 cell lines tested by CCK8 assay were 26.94 and 9.56 uM, respectively. Corresponding IC_50_ values of doxorubicin were 0.425 and 0.458 uM. Combination of the two drugs synergistically inhibited cell viability (Fig. 6A and B) with CI values < 1 (Fig. 6C and D). The results were confirmed by EdU assay. As shown in Fig. 6E and F, after 48 h of erastin and/or doxorubicin treatment, the number of EdU-positive cells in co-treatment group was significantly reduced than that in erastin or doxorubicin monotherapy group, which indicated a significant inhibition of DNA synthesis by the combination. Flow cytometry also showed an increasing cell death with co-treatment than monotherapies (Fig. 7A and B). ROS production is the pivotal cytotoxic mechanism of ferroptosis. As the results shown by fluorescence microplate reader (Fig. 8A and B), either erastin or doxorubicin may promote the generation of intracellular ROS in MM cell lines. However, combination of the two drugs significantly stimulated the accumulation of ROS. Similar results were observed by fluorescence microscopy (Fig. 8C and D). We detected the protein level of several ferroptosis-related markers to investigate the potential molecular mechanism between the drug combination, ROS production and ferroptosis. The protein level of GPX4 and Keap1 was significantly decreased while the SLC7A11 protein level was increased in MM cell lines after treated with erastin and doxorubicin (Fig. 8E and F). Furthermore, changes of RAS signaling pathway and intracellular ATP levels were detected. An increase of Raf protein level while a decrease of ERK and KRAS protein level (Additional file 5: Figure S4A and B), and an elevated ATP level (Additional file 5: Figure S4C and D) were observed after drug(s) administration.

### Other small-molecule gene inhibitors sensitizing MM cell lines to doxorubicin

G6PD and NQO1 are ferroptosis-related genes that also associate with poor survival in the gene model. Polydatin is a small-molecule inhibitor for G6PD, and dicoumarol is an inhibitor for NQO1. Both polydatin and dicoumarol were observed to sensitize the inhibitory proliferation effect of doxorubicin in MM cell lines (Additional file 6: Figure S5).

## Discussion

MM is a common hematological malignancy. Various factors including patient physical condition, tumor biological behavior, cytogenetic abnormalities and gene expression profile were associated with survival outcomes of MM patients [23, 24]. The widely recognized prognostic models currently ISS and RISS have considered the relation of many crucial co-variates with patient survival. However, there were only few studies on the prognostic impact of gene expression profiles in MM at present.

Ferroptosis is a type of cell death mediated by iron-dependent peroxidation, which has been demonstrated to exert antitumor effect in multiple malignancies [25, 26]. In this study, we incorporated FRGs that associated with MM survival to develop a prognostic model, which was more accurate in survival prediction than ISS. Furthermore, the gene expression profile of MM involved in this study provided an upfront foundation and promising targets for the exploration of novel drugs. ATG7, HMOX1, LPIN1, MAPK8 and TF were positively while AURKA, FH, G6PD, NQO1, TXNRD1, VDAC2 were negatively correlated with survival. Particularly, VDAC2 was highly expressed in tumor tissues relative to normal tissues (Additional file 2: Figure S1). VDAC2 is a component of voltage-dependent anion channels (VDAC) on the outer membrane of mitochondria and plays an important role in cell metabolism and cell death by regulation of the substance exchanges between cytoplasm and mitochondria [27]. The ferroptosis inducer erastin works by binding to VDAC2, keeping VDAC opening and altering mitochondrial membrane permeability, resulting in the increase of mitochondrial metabolism and ROS production [28].

Doxorubicin is a classical chemotherapy agent for the treatment of MM. Combination with effective small-molecule drugs would help enhance the cytotoxic effect of low-dose doxorubicin against tumor cells, exerting desirable antitumor effects and alleviating the toxic side effects of doxorubicin [29]. Several studies have shown that doxorubicin alone or combination with other agents was able to induce production of ROS [30, 31]. Our in vitro experiments have proved the synergistic effect of erastin and doxorubicin against MM cell lines. The underlying mechanism is the overgeneration of ROS with much higher intracellular ROS level in the combination group.

Additionally, the molecular mechanisms have also been preliminarily studied. Erastin was shown to inactivate Glutathione peroxidase 4 (GPX4) by inhibiting System Xc- and GSH production [32]. GPX4 was identified as a core ferroptosis regulator that maintains membrane lipid layer homeostasis by reducing lipid peroxide toxicity. Inactivation of GPX4 results in the accumulation of intracellular peroxides, production of ROS and triggering ferroptosis [12, 32]. The significantly decreasing protein level of GPX4 after supplement of erastin and doxorubicin may be one of the probable molecular mechanisms for ROS accumulation and ferroptosis in this study. Indeed, erastin induced a decrease of Keap1 protein level in our experiments, which was consistent with previous study [33], and drug combination seemed to exacerbate the change. Sun et.al explained an increasing interaction of p62 and Keap1 after treating hepatocellular carcinoma cells with erastin [33], but the inherent mechanism of our study needs further investigation. An increasing SLC7A11 protein level after drug treatment could be interpreted as an adaptive reaction to the suppression of system Xc^−^ by erastin [34, 35].

It has been reported that erastin keeps the opening of VDAC to exacerbate the influx of respiratory subjects into the mitochondria, which increases mitochondrial metabolism and generates abundant ATP to inhibit glycolysis [36]. The elevated intracellular ATP level preliminarily indicated an increasing mitochondrial metabolism and oxidative phosphorylation (OXPHOS) driven by erastin treatment in our study.

There still exist several limitations in our study. First, the data for the prognostic model were retrospectively retrieved from public databases, which needs to be further verified by evidence from real-world and prospective studies. Second, there is an inevitable intrinsic disadvantage in predicting survival by only one signature related prognostic gene model, and genes involving other characteristics are needed to refined the model. Next, further mechanisms such as changes in RAS signaling pathway and OXPHOS level caused by erastin still need to be studied in the future. Finally, the results of in vitro experiments also need to be confirmed by in vivo experiments.

To sum up, we developed a prognostic model for MM including 11 FRGs, which exhibited great prognostic accuracy in both training and validation cohorts. Moreover, a synergistic effect of the ferroptosis inducer erastin and the classical chemotherapeutic agent doxorubicin was illustrated in vitro. These results indicated ferroptosis could serve as a measurement in survival prediction and an antitumor target for MM, which would assist in patient diagnosis and prognosis, and design of clinical trials. Furthermore, the study also provided a preliminary foundation for mechanism research on ferroptosis.

## Supplementary Information

**Additional file 1: Table S1.** 11 genes identified by Lasso regression analysis for the prognostic model.

**Additional file 2: Figure S1.** Differential expression of the 11 genes between normal and MM tissues in the (A) GSE6477 and the (B) GSE118985 datasets.

**Additional file 3: Figure S2.** Genetic alterations of the 11 FRGs in CCLE.

**Additional file 4: Figure S3.** The relationship between clinical characteristics and risk scores in the training cohort.

**Additional file 5: Figure S4.** Changes in RAS signaling pathway (A and B) and intracellular ATP level (C and D) after drug(s) treatment by invitro experiments.

**Additional file 6: Figure S5.** Polydatin and dicoumarol sensitized MM cell lines to doxorubicin. Cell viabilities measured by CCK8 assay after MM cell lines treated with (A) doxorubicin or doxorubicin plus 60uM polydatin; (B) doxorubicin or doxorubicin plus 30uM dicoumarol for 48h.

## Data Availability

The datasets analyzed by the study were available in public databases that could be acquired on http://www.ncbi. nlm.nih.gov/geo/.
