# Supplementary material for: Integrated assessment of the clinical and biological value of ferroptosis-related genes in multiple myeloma
Source: Cancer Cell Int. 2022 Oct 23;22:326. doi: 10.1186/s12935-022-02742-4 (PMC9588243; doi:10.1186/s12935-022-02742-4)
Supplement: Supplementary file 1 — Additional file 1: Table S1. 11 genes identified by Lasso regression analysis for the prognostic model. [file 12935_2022_2742_MOESM1_ESM.docx]

| **Gene** | **Coefficient** |
| --- | --- |
| ATG7 | -0.4358 |
| AURKA | 0.3907 |
| FH | 0.1889 |
| G6PD | 0.3740 |
| HMOX1 | -0.0624 |
| LPIN1 | -0.2988 |
| MAPK8 | -0.4663 |
| NQO1 | 0.1110 |
| TF | -0.5460 |
| TXNRD1 | 0.0926 |
| VDAC2 | 0.2743 |

**Table S1.** 11 genes identified by Lasso regression analysis for the prognostic model.
