# Supplementary figures and images for: Integrated assessment of the clinical and biological value of ferroptosis-related genes in multiple myeloma
Source: Cancer Cell Int. 2022 Oct 23;22:326. doi: 10.1186/s12935-022-02742-4 (PMC9588243; doi:10.1186/s12935-022-02742-4)

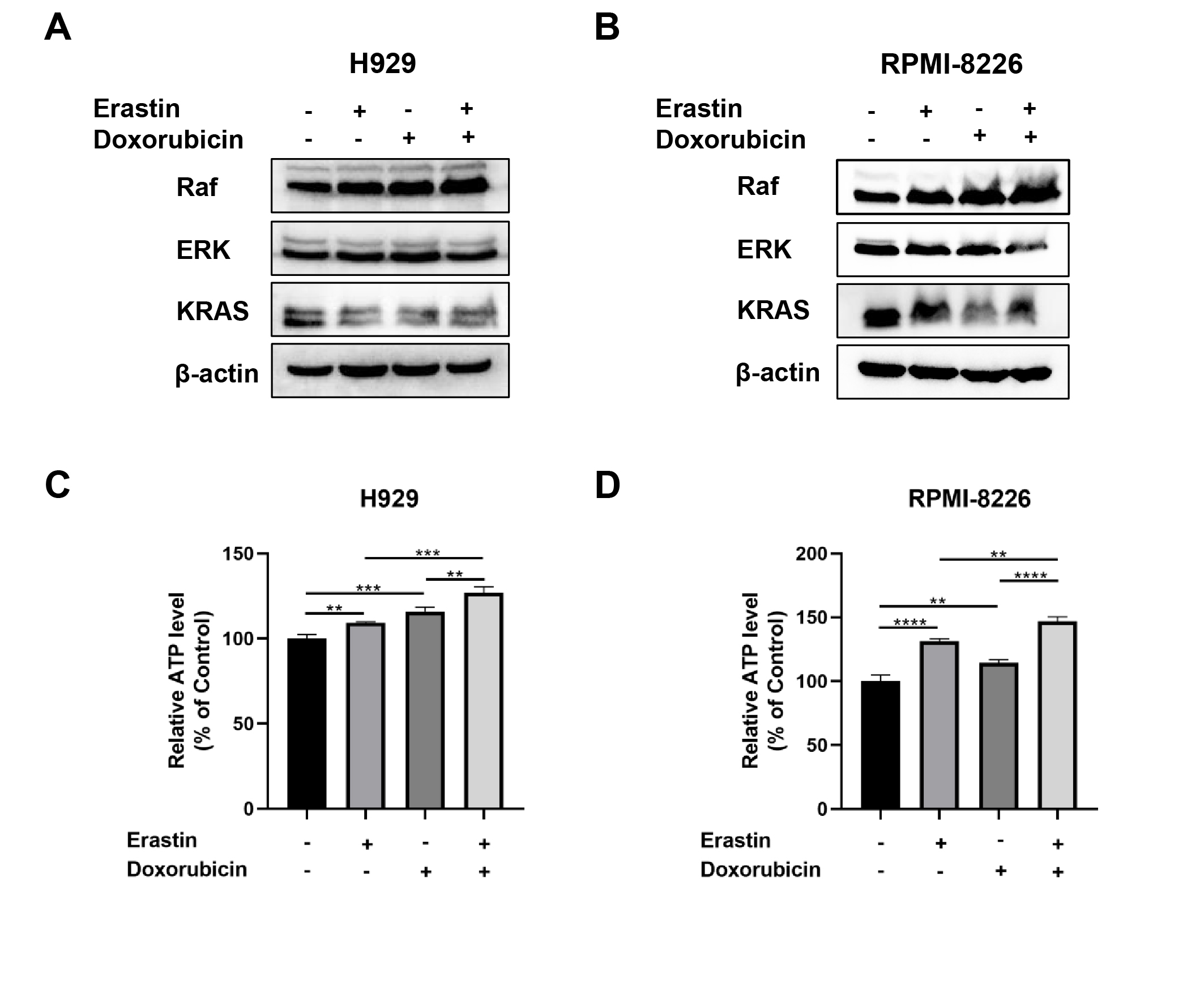

Supplement: Supplementary file 5 — Additional file 5: Figure S4. Changes in RAS signaling pathway (A and B) and intracellular ATP level (C and D) after drug(s) treatment by invitro experiments. [file 12935_2022_2742_MOESM5_ESM.jpg]

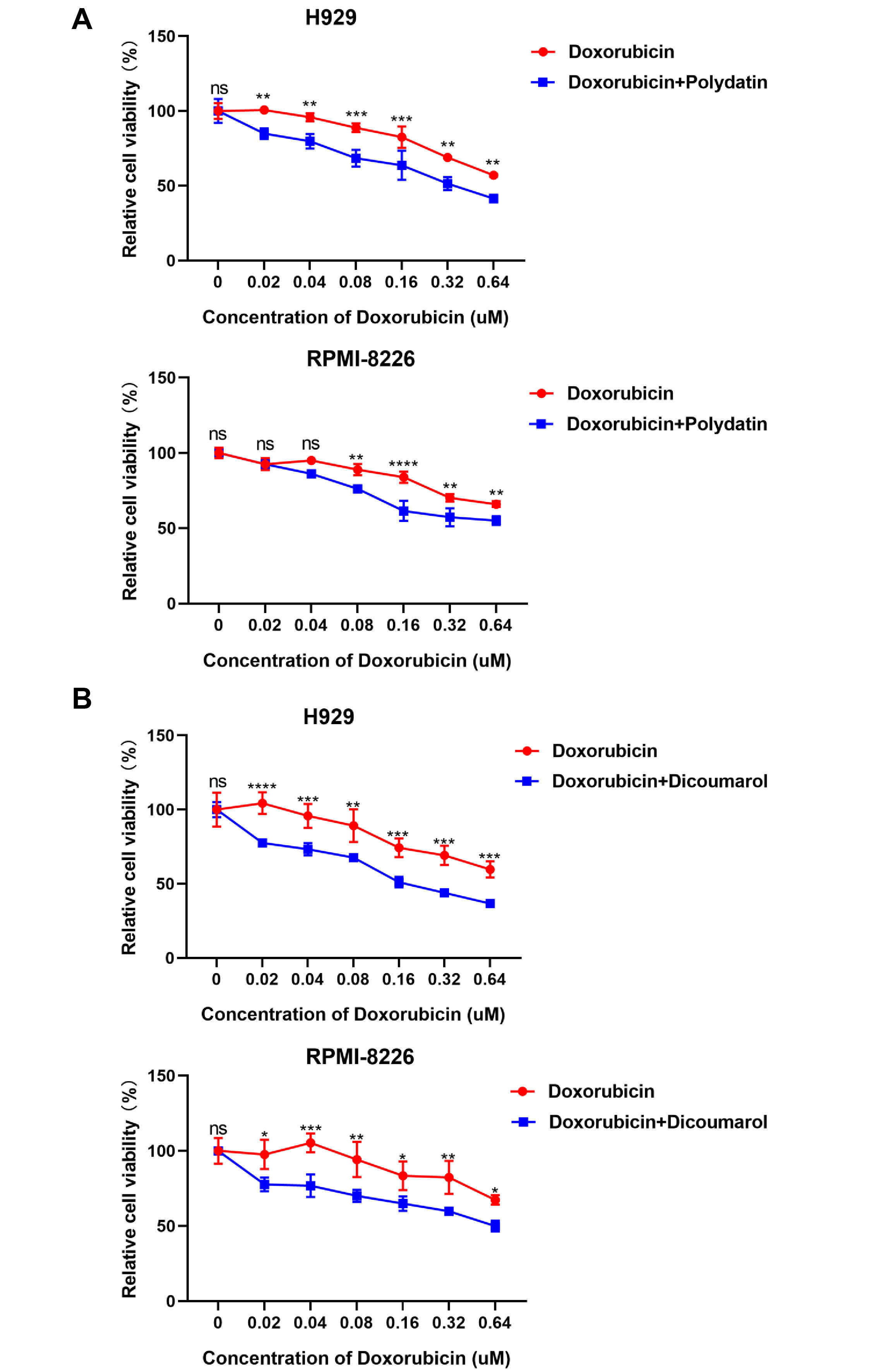

Supplement: Supplementary file 6 — Additional file 6: Figure S5. Polydatin and dicoumarol sensitized MM cell lines to doxorubicin. Cell viabilities measured by CCK8 assay after MM cell lines treated with (A) doxorubicin or doxorubicin plus 60uM polydatin; (B) doxorubicin or doxorubicin plus 30uM dicoumarol for 48h. [file 12935_2022_2742_MOESM6_ESM.jpg]
